# Zinc induces epithelial to mesenchymal transition in human lung cancer H460 cells via superoxide anion-dependent mechanism

**DOI:** 10.1186/s12935-016-0323-4

**Published:** 2016-06-17

**Authors:** Chuanpit Ninsontia, Preeyaporn Plaimee Phiboonchaiyanan, Pithi Chanvorachote

**Affiliations:** Department of Pharmacology and Physiology, Faculty of Pharmaceutical Sciences, Chulalongkorn University, Pathumwan, Bangkok, 10330 Thailand; Cell-based Drug and Health Products Development Research Unit, Faculty of Pharmaceutical Sciences, Chulalongkorn University, Bangkok, Thailand

**Keywords:** Zinc, Epithelial-to-mesenchymal transition, Superoxide anion, Lung cancer, Metastasis, Reactive oxygen species

## Abstract

**Background:**

Epithelial to mesenchymal transition (EMT) has been shown to be a crucial enhancing mechanism in the process of cancer metastasis, as it increases cancer cell capabilities to migrate, invade and survive in circulating systems. This study aimed to investigate the effect of essential element zinc on EMT characteristics in lung cancer cells.

**Methods:**

The effect of zinc on EMT was evaluated by determining the EMT behaviors using migration, invasion and colony formation assay. EMT markers were examined by western blot analysis. Reactive oxygen species (ROS) were detected by specific fluorescence dyes and flow cytometry. All results were analyzed by ANOVA, followed by individual comparisons with post hoc test.

**Results:**

The present study has revealed for the first time that the zinc could induce EMT and related metastatic behaviors in lung cancer cells. Results showed that treatment of the cells with zinc resulted in the significant increase of EMT markers N-cadherin, vimentin, snail and slug and decrease of E-cadherin proteins. Zinc-treated cells exhibited the mesenchymal-like morphology and increased cancer cell motility with significant increase of activated FAK, Rac1, and RhoA. Also, tumorigenic abilities of lung cancer cells could be enhanced by zinc. Importantly, the underlying mechanism was found to be caused by the ability of zinc to generate intracellular superoxide anion. Zinc was shown to induce cellular superoxide anion generation and the up-regulation of EMT markers and the induced cell migration and invasion in zinc-treated cells could be attenuated by the treatment of MnTBAP, a specific superoxide anion inhibitor.

**Conclusion:**

Knowledge gains from this study may highlight the roles of this important element in the regulation of EMT and cancer metastasis and fulfill the understanding in the area of cancer cell biology.

## Background

Metastatic potential of lung cancer cells has been accepted to be an important cause of high rate of death worldwide [1]. Knowledge indicates that the process of cancer cell transition from epithelial to mesenchymal phenotypes or epithelial to mesenchymal transition (EMT) plays a dominate role in facilitating metastasis and progression in many types of cancer [2–4]. EMT-phenotypic cancer cells elicit highly metastatic potentials, such as aggressive migratory, invasive and increased tumorigenicity [2–5]. During EMT, epithelial cells undergo remarkable morphological conversion from stone-like epithelial morphology to elongated-like mesenchymal morphology and the crucial hallmarks of EMT are the loss of E-cadherin, a cellular junction protein typically expressed in epithelial cells, and the increase of mesenchymal markers (e.g. N-cadherin, vimentin, snail and slug) [2–5]. Zinc is a trace element implicated in many important cellular processes including structural, functional and signaling of the cells [6, 7]. Zinc is detected in plasma at the concentrations ranging from 10 to 18 µM [6, 7] and the concentration of zinc in plasma or tissues is found to be elevated in pathological condition of cancer [8–11]. Importantly, evidence indicates that several zinc influx transporters such as Zrt/Irt-like protein (ZIP) 6 [12], ZIP7 [13] and ZIP10 [14] were shown to be up-regulated in cancer cells and their high levels correlate with aggressive behaviors and poor prognosis. Such data has suggested the roles of zinc in regulation of cancer cell biology. However, effects of zinc on the EMT process of cancer cells are largely unknown.

Recently, the involvement of ROS in EMT process has been continuously revealed [15–17]. Together with the fact that zinc has been shown to regulate cellular redox status of the cells [18–20], it is possible that zinc may affect the cancer cell behaviors via ROS-dependent mechanism. Indeed, the exogenous zinc has a capability to induce ROS production via NADPH oxidase and mitochondria-dependent mechanism [18–20]. Zinc exposure was found to induce translocation of NADPH oxidase subunits to plasma membrane, which is the signature event for NADPH oxidase activation and such an event was inhibited by the addition of NADPH oxidase inhibitor [7, 19, 20]. Together, we hypothesize that zinc may affect the process of EMT in lung cancer cells. Also, we attempt to clarify the mechanisms involved in zinc-induced EMT. The findings from this study could help fulfill the understanding in tumor cell biology and could provide important information useful for zinc management in cancer patients.

## Methods

### Cells and reagents

Human lung cancer epithelial H460 cell was obtained from the American Type Culture Collection (ATCC, Manassas, VA). H460 cell was cultured in RPMI 1640 medium in a 5 % CO_2_ environment at 37 °C. The media was supplemented with 2 mM l-glutamine, 10 % fetal bovine serum and 100 units/ml of penicillin/streptomycin (Gibco, Gaithersburg, MA, USA). Zinc sulfate, dimethyl sulfoxide (DMSO), 2,7-dichlorofluorescein diacetate (DCFH_2_-DA), dihydroethidium (DHE), hydroxyphenyl fluorescein (HPF), DMNQ (2,3-dimethoxy-1,4-naphthoquinone),3-(4,5-Dimethylthiazol-2-yl)-2,5-diphenyltetrazolium bromide (MTT) and Hoechst 33342 were obtained from Sigma Chemical, Inc. (St. Louis, MO, USA). Mn (ΙΙΙ) tetrakis (4-benzoic acid) porphyrin chloride (MnTBAP) was obtained from Calbiochem (San Diego, CA, USA). Antibodies for N-cadherin, E-cadherin, vimentin, snail, slug, phosphorylated FAK (Y397), FAK, and β-actin and peroxidase-labeled secondary antibodies were obtained from Cell Signaling Technology, Inc. (Denver, MA). Mouse monoclonal antibodies for active Rho-GTP and Rac1-GTP were obtained from NewEast Biosciences (Malvern, PA, USA). Immobilon Western chemiluminescent HRP substrate was obtained from Millipore, Corp (Billerica, MA, USA) and Thermo Fisher Scientific Inc. (Rockfort, IL, USA).

### Cytotoxicity assay

Cell viability was determined by MTT colorimetric assay. Briefly, cells in 96-well plate were incubated with 500 μg/ml of MTT for 4 h at 37 °C. The supernatant was then removed and dimethylsulfoxide (DMSO) was added to dissolve the formazan product. The intensity was spectrophotometrically measured at 570 nm using an ELISA reader (Anthros, Durham, NC, USA). All analyses were performed in at least three independent replicate cultures. The optical density ratio of treated to non-treated control cells was calculated and presented in terms of relative cell viability.

### Apoptosis assay

Apoptotic cell death was detected by Hoechst 33342 staining. After specific treatments, cells were stained with 10 µM of the Hoechst 33342 for 30 min at 37 °C. The apoptotic cells having condensed chromatin and/or fragmented nuclei stained by Hoechst 33342 were visualized and scored under a fluorescence microscope (Olympus IX51 with DP70).

### Cell morphology characterization

Cell morphology was investigated by seeding the cells at a density of 5 × 10^4^ cells/well onto a 12-well plate for 48 h. The cells were treated with various concentrations of zinc sulfate for 24 h. The cells were then washed with PBS, fixed with 4 % paraformaldehyde in PBS for 10 min at 37 °C, rinsed three times with PBS, and mounted with 50 % glycerol. Cell morphology was then assessed by a phase contrast microscope (Eclipse Ti-U, Nikon, Tokyo, Japan).

### Immunofluorescence

Cells were seeded at a density of 1 × 10^5^ cells/well onto coverslips in six-well plate and incubated overnight. After the treatment, the cells on coverslips were fixed with 4 % paraformaldehyde for 30 min and permeabilized with 0.1 % Triton-X for 20 min. Thereafter, the cells were incubated with 3 % bovine serum albumin (BSA) for 30 min to prevent nonspecific binding. The cells were washed and incubated with rabbit anti-Vimentin antibody for 1 h at room temperature. Primary antibody was removed and the cells were washed and subsequently incubated with Alexa Fluor 488 (Invitrogen) conjugated goat anti-rabbit IgG (H + L) secondary antibody for 1 h at room temperature. Samples were washed with PBS then visualized and imaged by fluorescence microscope (Olympus IX 51 with DP70, Olympus America Inc., Center valley, PA).

### Western blot analysis

After specific treatments, cells were incubated in lysis buffer containing 20 mM Tris–HCl (pH 7.5), 1 % Triton X-100, 150 mM sodium chloride, 10 % glycerol, 1 mM sodium orthovanadate, 50 mM sodium fluoride, 100 mM phenylmethylsulfonyl fluoride, and a protease inhibitor cocktail (Roche Molecular Biochemicals) for 90 min on ice. The cell lysates were collected, and the protein content was determined using the BCA protein assay kit (Thermo scientific, IL, USA). Equal amounts of proteins from each sample (60 μg) were denatured by heating at 95 °C for 5 min with Laemmli loading buffer and subsequently loaded onto a 10 % SDS-PAGE. After separation, proteins were transferred onto 0.45 μM nitrocellulose membranes (Bio-Rad, Hercules, CA). The transferred membranes were blocked for 1 h in 5 % nonfat dry milk in TBST (25 mM Tris–HCl pH 7.5, 125 mM NaCl, and 0.05 % Tween 20) and incubated with the appropriate primary antibodies at 4 °C overnight. Then, the membranes were washed twice with TBST for 10 min and incubated with horseradish peroxidase-labeled isotype-specific secondary antibodies for 2 h at room temperature. The immune complexes were detected by enhancement with chemiluminescence substrate (Supersignal West Pico; Pierce, Rockfore, IL) and quantified the level of proteins using imageJ software.

### Migration assay

Migration was determined by wound healing and transwell assays. For the wound healing assay, a monolayer of cells was cultured in a 96-well plate, and a wound space was made with a 1-mm-wide tip. After rinsing with PBS, the cell monolayers were incubated with the indicated treatments and allowed to migrate for 24 h. Micrographs were taken under a phase contrast microscope (Olympus DP70, Melville, NY), and the wound spaces were measured using Olympus DP controller software. Quantitative analysis of cell migration was performed using an average wound space from those random fields of view, and the percentage of change in the wound space was calculated using the following formula: % change = (average space at time 0 h) − (average space at time 24 h)/(average space at time 0 h) × 100. Relative cell migration was calculated by dividing the percentage change in the wound space of treated cells by that of the control cells in each experiment. For the transwell assay, the cells were seeded at a density of 5 × 10^4^ cells/well onto the upper chamber of a transwell (8 µm pore size) in a 24-well plate in serum-free medium and incubated with various concentrations of zinc. RPMI medium containing 10 % FBS was added to the lower chamber. Following the incubation, the non-migrated cells in the upper chamber were removed by cotton-swab wiping, and the cells that migrated to the underside of the membrane were stained with 10 µg/ml of Hoechst 33342 for 10 min and visualized and scored under a fluorescence microscope (Olympus IX51 with DP70).

### Invasion assay

An invasion assay was performed using a 24-well transwell unit with polycarbonate (PVDF) filters (8 µm pore size). The membrane was coated with 0.5 % matrigel on the upper surface of the chamber overnight at 37 °C in a humidified incubator. The cells were plated at a density of 2 × 10^4^ cells per well into the upper chamber of the transwell unit in serum-free medium. Medium containing 10 % FBS was added to the lower chamber of the unit. After incubation with specific test agents for 24 h at 37 °C, the medium in the upper chamber was aspirated, and the cells on the upper side of the membrane were removed with a cotton swab. The cells that invaded to the underside of the membrane were stained with 10 µg/ml of Hoechst 33342 for 10 min, visualized and scored under a fluorescence microscope (Olympus IX51 with DP70).

### In vitro 3D tumorigenesis assay

In vitro 3D tumorigenesis was performed in a matrigel-coated 96-well plate. A plate was coated with 0.5 % agarose and left for solidification. The cells were suspended in culture medium containing 4 % matrigel and various concentrations of zinc, and plated at a density of 3 × 10^2^ cells/well onto a agarose-coated plate. Medium containing various concentrations of zinc were replaced every 3 days. After 10 days, the cells were visualized and scored by image analyzer under microscope (Olympus IX51 with DP70). Whole area of each well was captured in one picture and the colonies with more than 25 µm of diameter were quantified.

### ROS detection

Intracellular ROS were determined by fluorescence microplate reader and by flow cytometry using the ROS-specific probe, superoxide anions, hydrogen peroxide and hydroxyl radicals were determined by DHE, DCFH_2_-DA and HPF, respectively. For fluorescence microplate reader, cells were seeded overnight in 96-well plate. Before zinc treatment cells were incubated with 10 μM of dihydroethidium (DHE), dichlorofluorescein diacetate (DCFH_2_-DA) or hydroxyphenyl fluorescein (HPF) for 30 min at 4 °C, after which they were washed and treated with various concentrations of zinc (0–50 µM) for 1 and 3 h. After incubation, the fluorescence intensity was immediately analyzed by fluorescence microplate reader (SpectraMax M5, Molecular Devices Corp., Sunnyvale, CA, USA) using a 488-nm excitation beam and a 610-nm band-pass filter for DHE, using a 480-nm excitation beam and a 530-nm band-pass filter for detecting DCF fluorescence or using a 490-nm excitation beam and a 515-nm band-pass filter for HPF. For flow cytometry, cells were seeded overnight in six-well plate. Before zinc treatment cells were incubated with 10 μM of DHE, DCFH_2_-DA or HPF for 30 min at 4 °C, after which they were washed and treated with 50 µM of zinc for 1 and 3 h. After incubation, cells were washed, re-suspended in phosphate-buffered saline (PBS), and immediately analyzed for fluorescence intensity by FACScan flow cytometer (Beckton Dickinson, Rutheford, NJ) using a 488-nm excitation beam and a 610-nm band-pass filter for DHE, using a 480-nm excitation beam and a 530-nm band-pass filter for detecting DCF fluorescence or using a 490-nm excitation beam and a 515-nm band-pass filter for HPF. Mean fluorescence intensity was quantified by CellQuest software (Becton–Dickinson) analysis of the recorded histograms. Relative fluorescence was calculated as a ratio of the treated to the non-treated control fluorescence intensity.

### Statistical analysis

All treatment data were normalized to non-treated controls. Data are expressed as the mean ± SD from three or more independent experiments. Multiple comparisons were examined for significant differences of multiple groups, using analysis of variance (ANOVA), followed by individual comparisons with post hoc test. Statistical significance was set at p < 0.05.

## Results

### Effects of zinc on viability of human lung cancer H460 cells

**Fig. 1:**
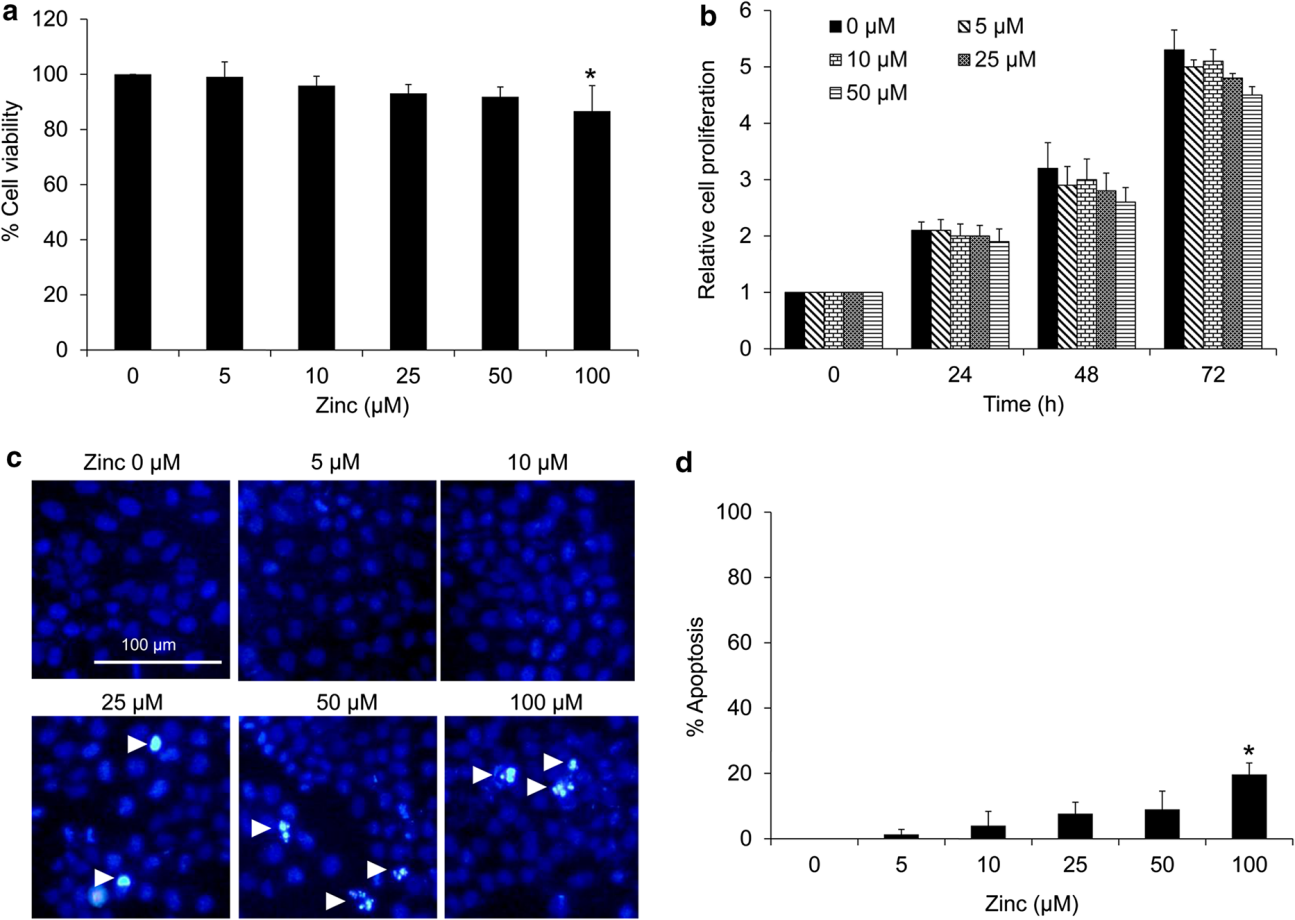
The cytotoxic and proliferative effects of zinc on lung cancer H460 cells. **a** Effect of zinc on cell viability. Cells were treated with various concentrations of zinc (0–100 µM) for 24 h. Percentage of cell viability was determined by the MTT assay. Values are means of the independent triplicate experiments ± SD. *p < 0.05 versus non-treated control. **b** Proliferative effect of zinc on H460 cells. Cells were treated with zinc (0–50 µM) for 0–72 h and analyzed by MTT assay. **c** Apoptotic cells were detected by Hoechst 33342 staining after 24 h of zinc treatment. White arrow indicates the apoptotic cells having condensed chromatin and/or fragmented nuclei. Values are means of independent triplicate experiments ± SD. *p < 0.05 versus non-treated control. **d** Nuclear morphology of the cells stained with Hoechst 33342 and visualized under a fluorescence microscope; *scale bar* 100 µm

In order to investigate the effect of zinc on EMT phenotypes in human lung cancer cells, we first evaluated the non-cytotoxic concentrations of zinc. Cells were cultured in the presence or absence of zinc (0–100 µM) for 24 h, and cell viability was determined by MTT assay at 24 h. The results indicated that treatment of the cells with zinc at the concentrations ranging from 5 to 50 µM caused no significant cytotoxicity (Fig. 1a). The significant decrease in cell viability was first detected in response to 100 µM of zinc treatment with approximately 86 % of the cells remaining viable.

Proliferative effect of zinc at above concentrations was further evaluated by treating the cells with zinc for 0–72 h. Figure 1b indicates that zinc at the concentrations of 0–50 µM had no inductive effect on cell proliferation. To confirm the effect of zinc on cell toxicity, cells were similarly treated with zinc for 24 h, and apoptosis was evaluated by Hoechst 33342 staining assay. Figure 1c, d show that apoptotic cells containing condensed and/or fragmented nuclei were not detectable in response to zinc treatment at the concentrations of 5–50 µM.

### Zinc induces epithelial to mesenchymal transition in human lung cancer H460 cells

**Fig. 2:**
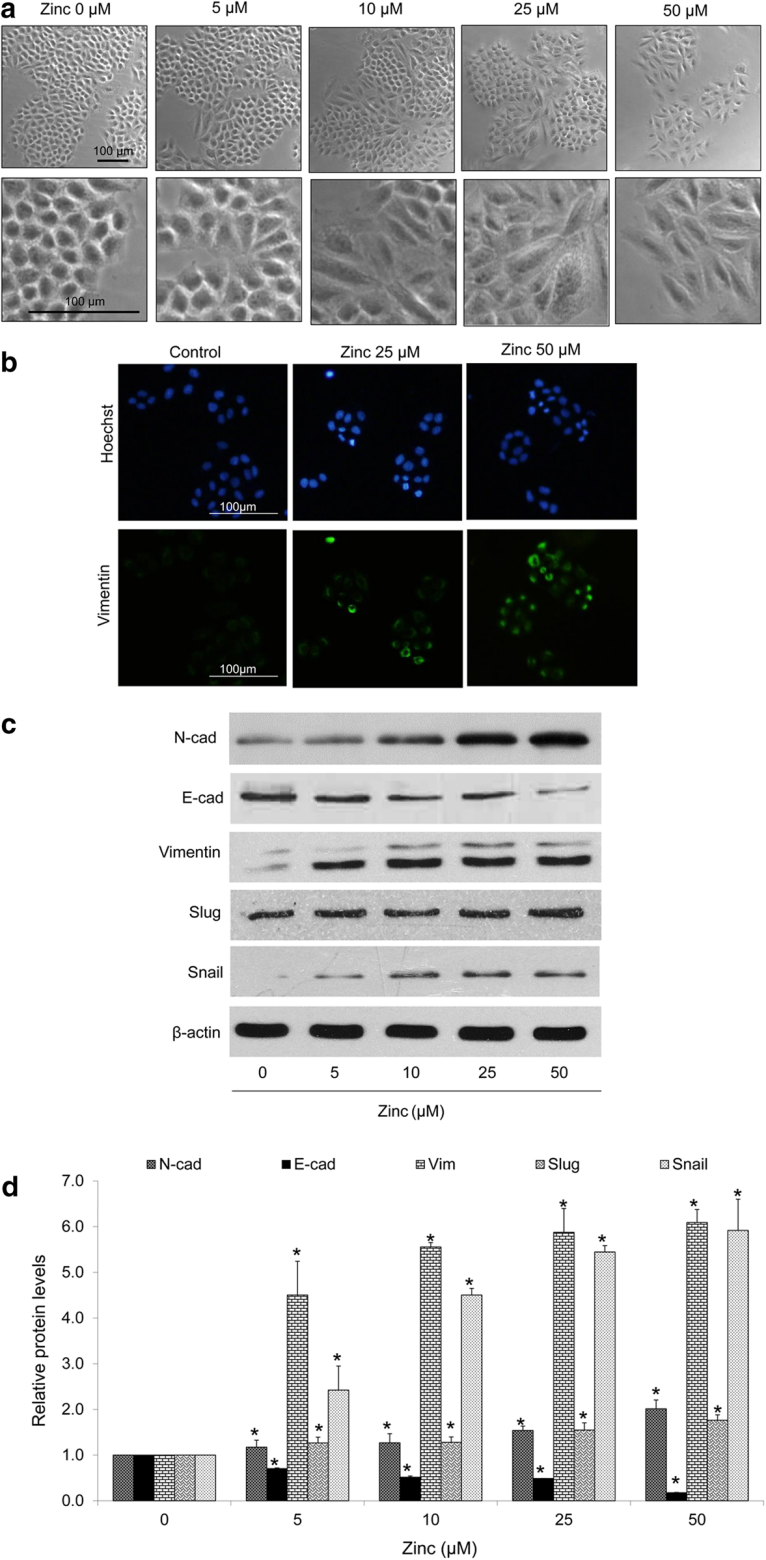
Effect of zinc on epithelial to mesenchymal transition (EMT). Cells were treated with various concentrations of zinc (0–50 µM) for 24 h. **a** Cells morphology was examined by phase-contrast microscope; *scale bar* 100 µm. **b** Expression of vimentin was analyzed by immunofluorescence staining; *scale bar* 100 µm. **c** The expression levels of EMT protein markers were determined by western blotting. The blots were re-probed with β-actin to confirm equal loading of the samples. **d** The blots were quantified by densitometry and mean data from three independent experiments were normalized to the results. The *bars* are the mean ± SD of independent triplicate experiments. *p < 0.05 versus untreated control

The effect of zinc on EMT in H460 cells was next investigated. The alteration of cell morphology as well as hallmarks of EMT were used to monitor the effect of zinc on EMT process in lung cancer cells. Cells were treated with zinc at non-toxic concentrations for 24 h. The morphology of the cells was captured and presented in Fig. 2a The results showed that the zinc-treated cells exhibited morphology of mesenchymal-like cells with the elongated shape and loss of cell polarity. These results also suggested that the mesenchymal-like morphology is somehow dose-dependent as the more elongated cells could be found in the cells treated with high concentrations of zinc. In addition, the expression of mesenchymal marker vimentin was significantly increased in response to zinc treatment (Fig. 2b).

The switch of E-cadherin to N-cadherin and increase of EMT proteins including vimentin, slug, and snail have been shown to be important hallmarks of EMT in cancer cells [2–5]. We next determined such cellular EMT markers in the lung cancer cells treated with zinc by western blot analysis. Obviously, treatment of the cells with zinc could reduce E-cadherin in a dose-dependent manner. Together with the fact that the significant increase of N-cadherin was found when treating the cells with 5–50 µM of zinc, these data strongly indicated that zinc could be able to mediate E-cadherin to N-cadherin switching in these cells. In addition, the upstream transcription factors of EMT namely snail and slug were determined in the zinc-treated cells. These factors were shown to bind to E-box elements in the promoter region of E-cadherin, resulting in the transcriptional repression of E-cadherin and induction of mesenchymal markers [2–4]. Figure 2c, d indicate that zinc significantly increased the levels of slug and snail. Also, the EMT protein vimentin was found to be induced by zinc. Taken together, our results suggested that zinc could induce EMT in lung cancer cells.

### Zinc facilitates H460 cell migration and invasion

**Fig. 3:**
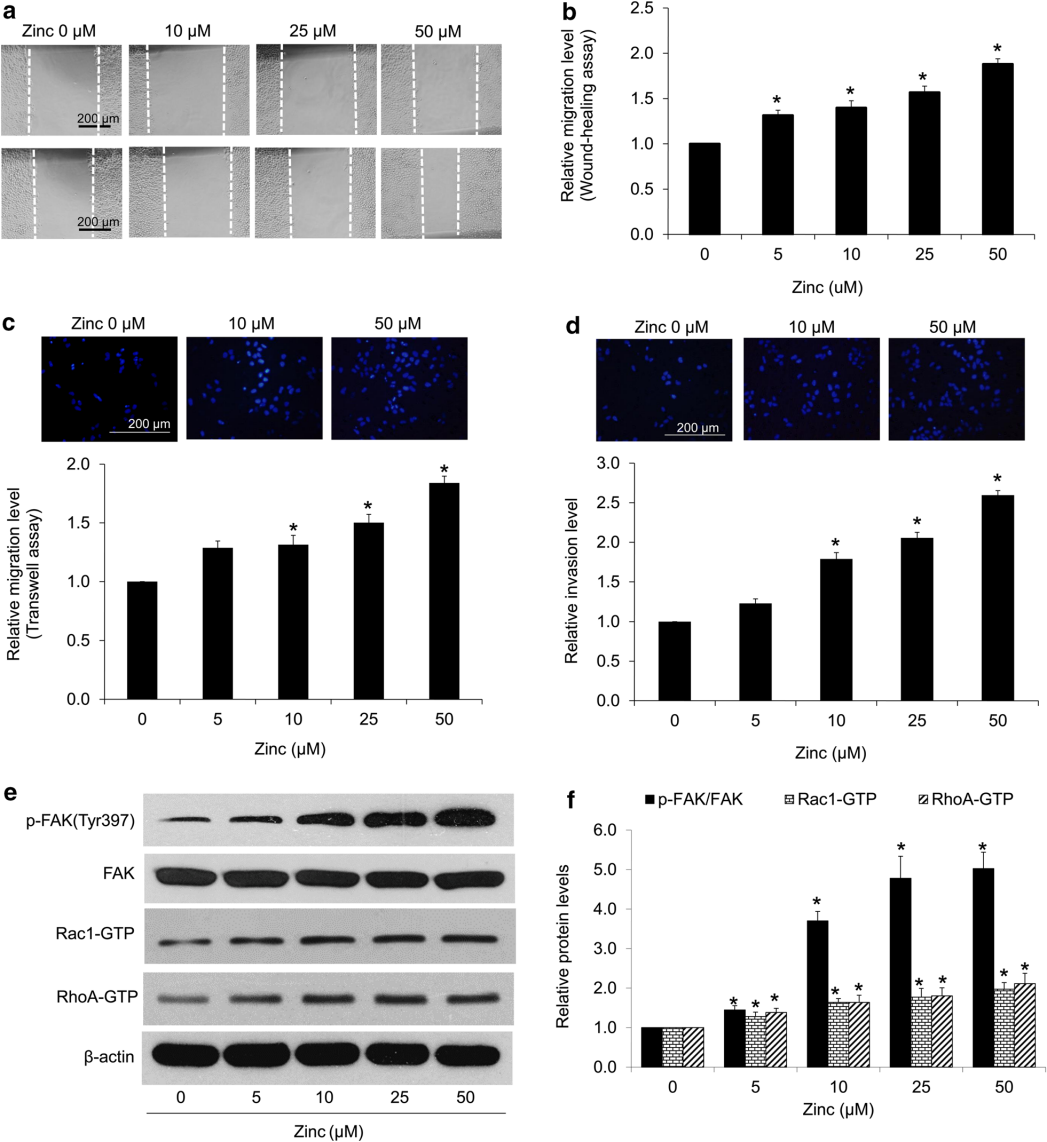
Effect of zinc on lung cancer cell migration and invasion. Cells were pre-treated with zinc (0–50 µM) for 24 h. The treated cells were subjected to migration and invasion assays. **a** For wound healing assay, the confluent monolayers of the cells were wounded by using a 1 mm-wide tip and cultured with the medium containing indicated treatments. After 24 h of incubation, wound spaces were analyzed and represented as a relative migration level. **b** The relative migration level was determined by comparing the relative change of zinc-treated cells to untreated control cells. Values are means of independent triplicate experiments ± SD. *p < 0.05 versus untreated control. **c** For transwell migration assay, migratory cells were stained with Hoechst 33342 for 30 min, determined under a fluorescence microscope and represented as average number of migratory cells in each field relatively to control cells. **d** Cell invasion was evaluated using a transwell coated with matrigel as described under “Methods” section. After 24 h, the cells that invaded across the membrane were stained with Hoechst 33342 for 30 min and visualized under a fluorescence microscope. Value was represented as average number of invaded cells in each field relatively to control. Values are means of independent triplicate experiments ± SD. *p < 0.05 versus untreated control. **e** The expression levels of motility-regulatory proteins were determined by western blotting. The blots were re-probed with β-actin to confirm equal loading of the samples. **f** The blots were quantified by densitometry and mean data from three independent experiments were normalized to the results. The bars are the mean ± SD of independent triplicate experiments. *p < 0.05 versus untreated control

One important phenotype of EMT cells is the increase in cell motility. Studies have demonstrated that EMT could enhance aggressiveness of tumor cells by increasing their ability to migrate and invade [2–4]. To evaluate the effect of zinc on cancer cell motility, cells were left untreated or pretreated with zinc at non-toxic concentrations for 24 h and subjected to migration and invasion assays as described in “Methods” section. Wound healing migratory assay showed that zinc significantly facilitated migratory activity of the cells with the relative cell migration increased approximately 1.3- to 1.8-fold in comparison to that of non-treated control cells (Fig. 3a, b). Also, the transwell migration assay was performed to confirm the migratory effect of zinc. Figure 3c shows that zinc treatment significantly increased the number of cells passed through the membrane of well, suggesting that such element induced cell migration.

Next, we performed experiments to test the ability of the cancer cells in invading through matrigel. The transwell was pre-coated with matrigel and the zinc-treated cells were seeded on top. The cells were allowed to invade for 24 h and the invaded cells at the lower part of the membrane were determined. Figure 3d shows that zinc significantly promoted the invasion of H460 cells in a dose-dependent manner.

Enhanced tumor cell migration and invasiveness are shown to be down-stream behaviors of FAK signal [21–24]. We next determined the effect of zinc treatment on motility regulatory proteins including FAK, activated (phosphorylated at Try397) FAK, active forms of RAC1, and RhoA by western blot analysis. The results showed that treatment of the cells with 0–50 µM of zinc for 24 h dramatically increased the activation of FAK (Fig. 3e, f). Also, its down-stream functioning proteins active Rac1 and RhoA were found to increase, accordingly. These results suggested that zinc treatment increase EMT-associated cell behaviors trough FAK-dependent pathway.

### Zinc enhances tumorigenicity in human lung cancer H460 cells

**Fig. 4:**
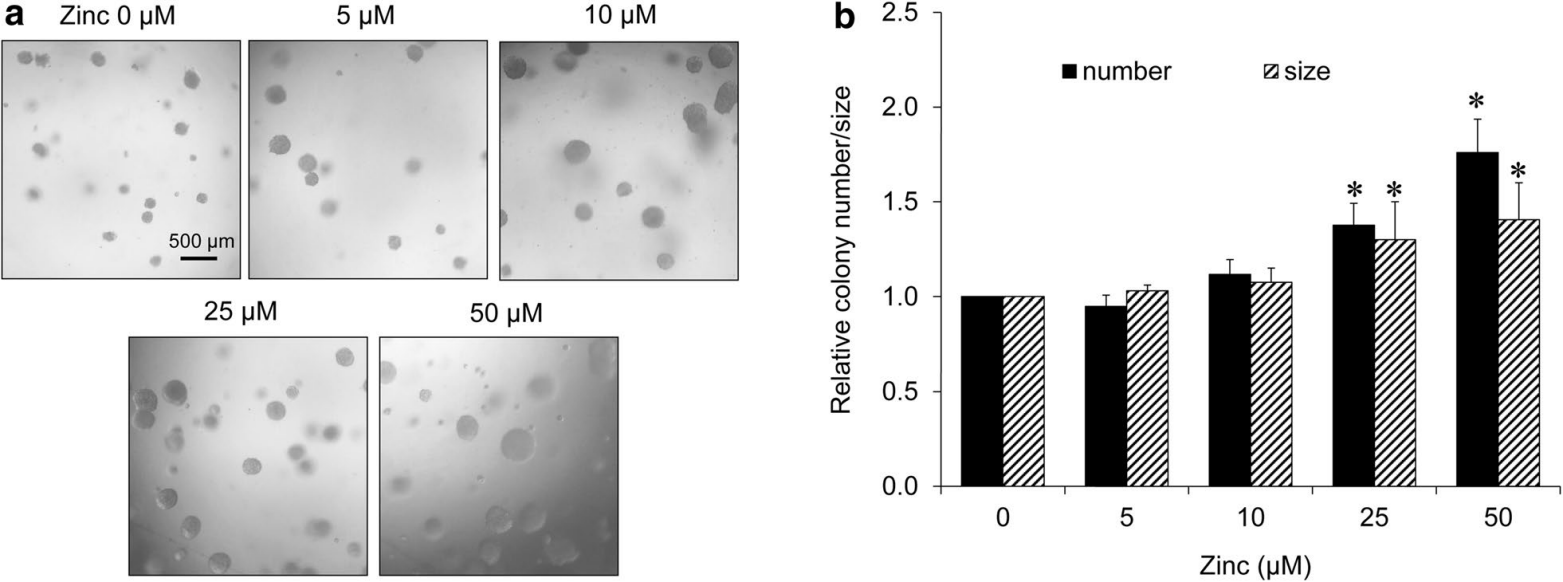
Zinc enhances tumorigenic potential of H460 lung cancer cells. **a** H460 cells were pre-treated with zinc (0–50 µM) for 24 h. The treated cells were subjected to 3D tumorigenesis assay. The cells were suspended in RPMI medium containing 4 % matrigel and zinc (0–50 µM) and plated onto agarose-coated plate. After 10 days, colonies were visualized under microscope; *scale bar* 500 µm. **b** Value was represented as average diameter and number of colonies in each field relatively to control cells using image analyzer. Values are means of independent triplicate experiments ± SD. *p < 0.05 versus untreated control

Having shown that zinc could enhance cell migration and invasion, we next tested whether zinc may augment the ability of cancer cell to initiate a new tumor. It is well known that the EMT process facilitate tumor formation at the metastatic site [25–27] and this potential is responsible for cancer progression [25–27]. Previous studies have shown that in vitro 3D tumorigenesis assay reflexes ability of the cancer cells in in vivo cancer condition [28]. Cells were left un-treated or pre-treated with various concentrations of zinc (0–50 µM) for 24 h and subjected to tumorigenesis assay as described in “Methods” section. After 10 days of 3D culturing, the colony number and diameter were determined. Figure 4 shows that zinc treatment enhances tumorigenic ability of the lung cancer cells as indicated by the significant increase in the number and size of colonies in zinc-treated groups. The number of colonies increased approximately 1.38- and 1.76-fold in response to zinc at 25 and 50 µM, respectively (Fig. 4b).

### Zinc induces intracellular superoxide anion generation in H460 cells

**Fig. 5:**
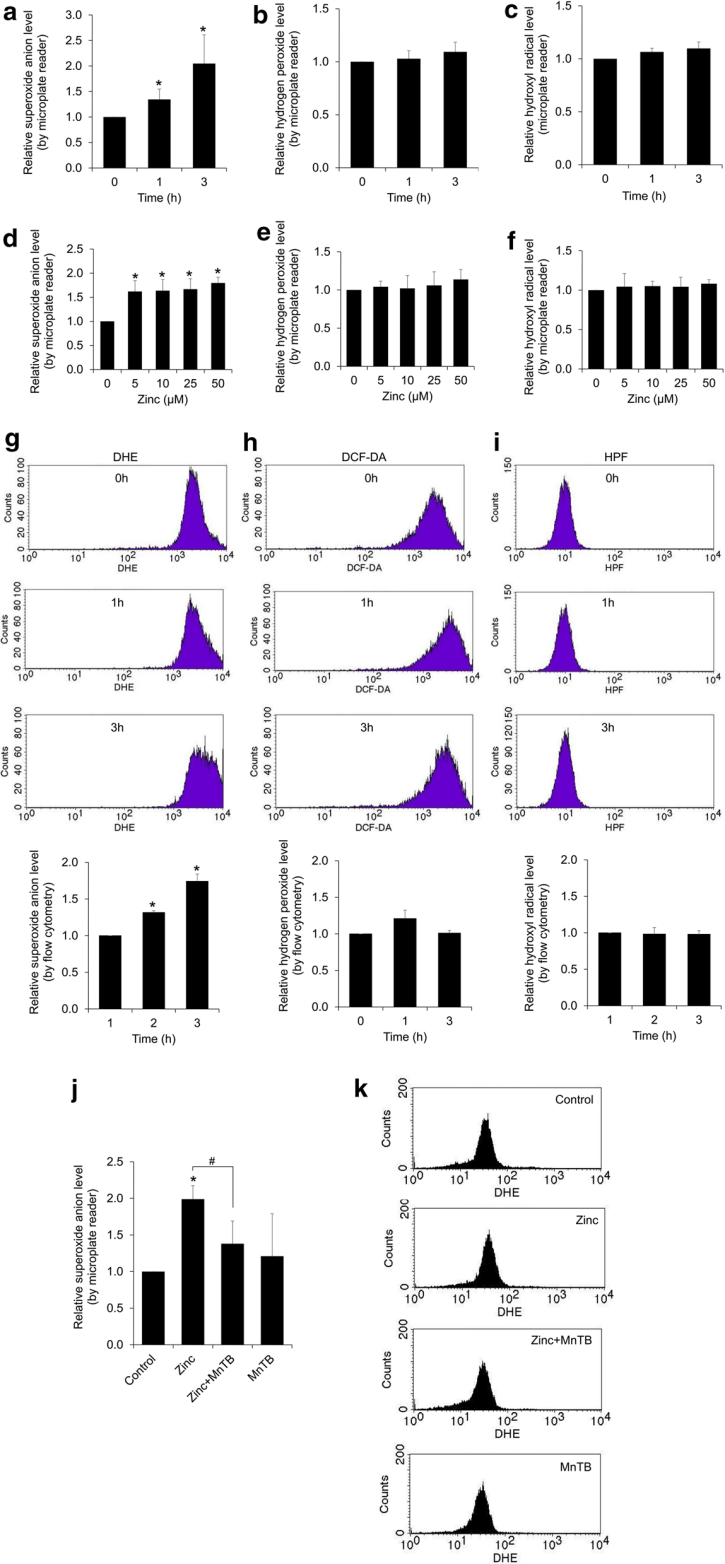
Zinc induces intracellular superoxide anion generation in H460 cells. **a**–**i** The cells were incubated with specific ROS probes, namely, DHE, DCFH_2_-DA, or HPF at 4 °C for 30 min prior to the treatment with zinc (0–50 µM) for 0–3 h and the fluorescence intensity was analyzed by a microplate reader and flow cytometry. Mean intensity was normalized to untreated control cells and represented as relative ROS levels. Values are means of independent triplicate experiments ± SD. *p < 0.05 versus untreated control. **j**–**k** The cells were incubated with DHE at 4 °C for 30 min prior to pre-treatment with 50 µM MnTBAP (superoxide anion inhibitor) for 1 h in the presence or absence of zinc (50 µM) for 3 h and the fluorescence intensity was analyzed by a microplate reader and flow cytometry. Mean intensity was normalized to untreated control cells and represented as relative ROS levels. Values are means of independent triplicate experiments ± SD. *p < 0.05 versus untreated control

Studies have demonstrated the roles of ROS on cancer EMT phenotypes [15–17]. Because zinc has been previously shown to affect the redox balance of the cells and exhibit the pro-oxidant activity in normal and cancer cells [7, 18–20], we next evaluated whether the effect of zinc in regulation of EMT in these lung cancer cells is through ROS-dependent mechanism. To examine the specific ROS levels induced by zinc, cells were incubated with various ROS specific probes as mentioned in “Methods” section for 30 min before treatment with 0–50 µM zinc and intracellular ROS levels were determined by flow cytometry and fluorescence microplate reader. Figure 5a, d show that zinc significantly increased cellular superoxide anion, while it had minimal effect on hydrogen peroxide (Fig. 5b, e) and hydroxyl radical (Fig. 5c, f). Consistent with microplate reader determination, flow cytometric analysis revealed that intracellular superoxide anion significantly increased up to twofold in response to zinc treatment (Fig. 5g) and zinc had no detectable effect on hydrogen peroxide and hydroxyl radical (Fig. 5h, i). We further confirmed the superoxide anion-inducing effect of zinc using specific superoxide anion inhibitor MnTBAP. Results indicated that treatment with the zinc caused the superoxide anion up-regulation in the cells and such event could be abolished by the addition of MnTBAP, confirming that the major ROS induced by zinc treatment in our system was superoxide anion (Fig. 5j, k).

### Zinc mediates EMT phenotypes in lung cancer cells through superoxide anion-dependent mechanism

**Fig. 6:**
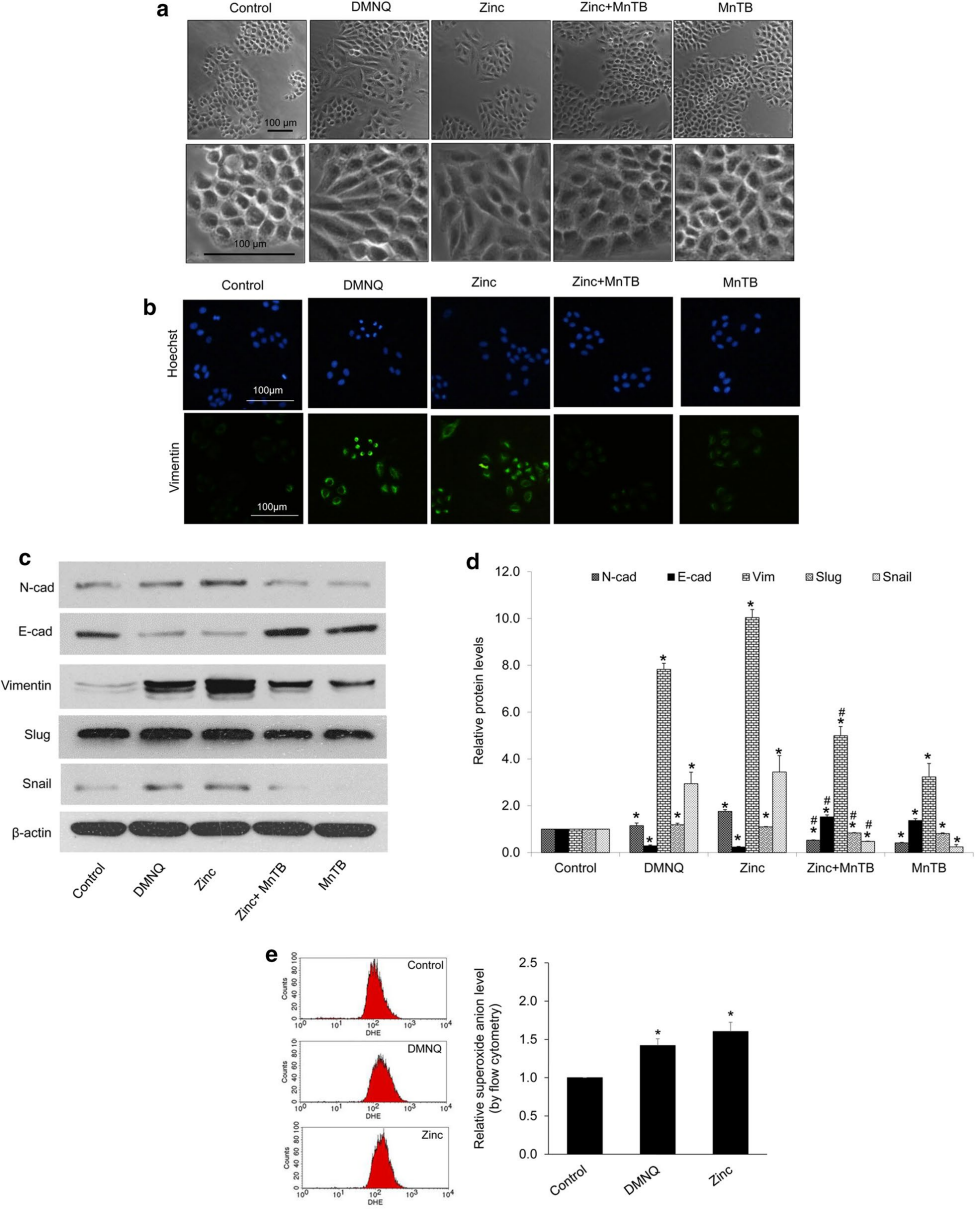
Effect of superoxide anion on EMT. **a** H460 cells were incubated with 5 µM DMNQ (superoxide anion inducer) or 50 µM MnTBAP (superoxide anion inhibitor), in the presence or absence of zinc (50 µM) for 24 h prior to morphology examination using phase contrast microscope; *scale bar* 100 µm. **b** Cells were treated with 5 µM DMNQ or 50 µM MnTBAP in the presence or absence of zinc (50 µM) for 24 h. Expression of vimentin were analyzed by immunofluorescence staining; *scale bar* 100 µm. **c** Cells were treated with 5 µM DMNQ or 50 µM MnTBAP, in the presence or absence of zinc (50 µM) for 24 h. The cells were collected and analyzed for EMT markers by western blotting. The blots were re-probed with β-actin to confirm equal loading. **d** The immunoblot signals were quantified by densitometry and mean data from independent experiments were normalized to the results. The *bars* are the mean ± SD of independent triplicate experiments. *p < 0.05 versus untreated control cells. ^#^p < 0.05 versus 50 µM zinc-treated cells. **e** Cells were incubated with DHE at 4 °C for 30 min prior to the treatment of DMNQ (5 µM) or zinc (50 µM), and the fluorescence intensity was analyzed by flow cytometry at 3 h. Mean intensity was normalized to untreated control cells and represented as relative superoxide anion levels. Values are means of independent triplicate experiments ± SD. *p < 0.05 versus untreated control

In order to investigate the role of superoxide anion on EMT induction, cell morphology, EMT markers, migratory behaviors, and colony formation were evaluated in the cells treated with zinc and specific inhibitor of superoxide anion. Cells were cultured in the presence or absence of MnTBAP for 1 h prior to zinc treatment and EMT phenotypes were determined. Also, to clarify role of superoxide anion, the superoxide anion inducer DMNQ was used. The results show that treatment of the cells with DMNQ or zinc alone was able to switch the morphology of lung cancer cells from epithelial to fibroblast-liked mesenchymal feature (Fig. 6a). Addition of MnTBAP to the zinc-treated cells could be able to attenuate such morphologic transformation.

Immunocytochemistry showed the increased vimentin found in DMNQ- and zinc- treated cells. And the increased vimentin signal induced by zinc was suppressed by the addition of MnTBAP (Fig. 6b). Consistently, the results of western blot analysis revealed that EMT markers including N-cadherin, vimentin, snail, and slug were found to be significantly increased in response to DMNQ and zinc treatment and such phenomenon could be reversed by the addition of MnTBAP (Fig. 6c, d).

As DMNQ was used as a superoxide anion donor, the intracellular superoxide anion generated by DMNQ was determined. Cells were incubated with DHE as mentioned in “Methods” section for 30 min and treated with 5 µM of DMNQ. DHE intensity was determined by flow cytometry. The result indicated the 1.5-fold superoxide induction in DMNQ-treated cells as shown in Fig. 6e.

**Fig. 7:**
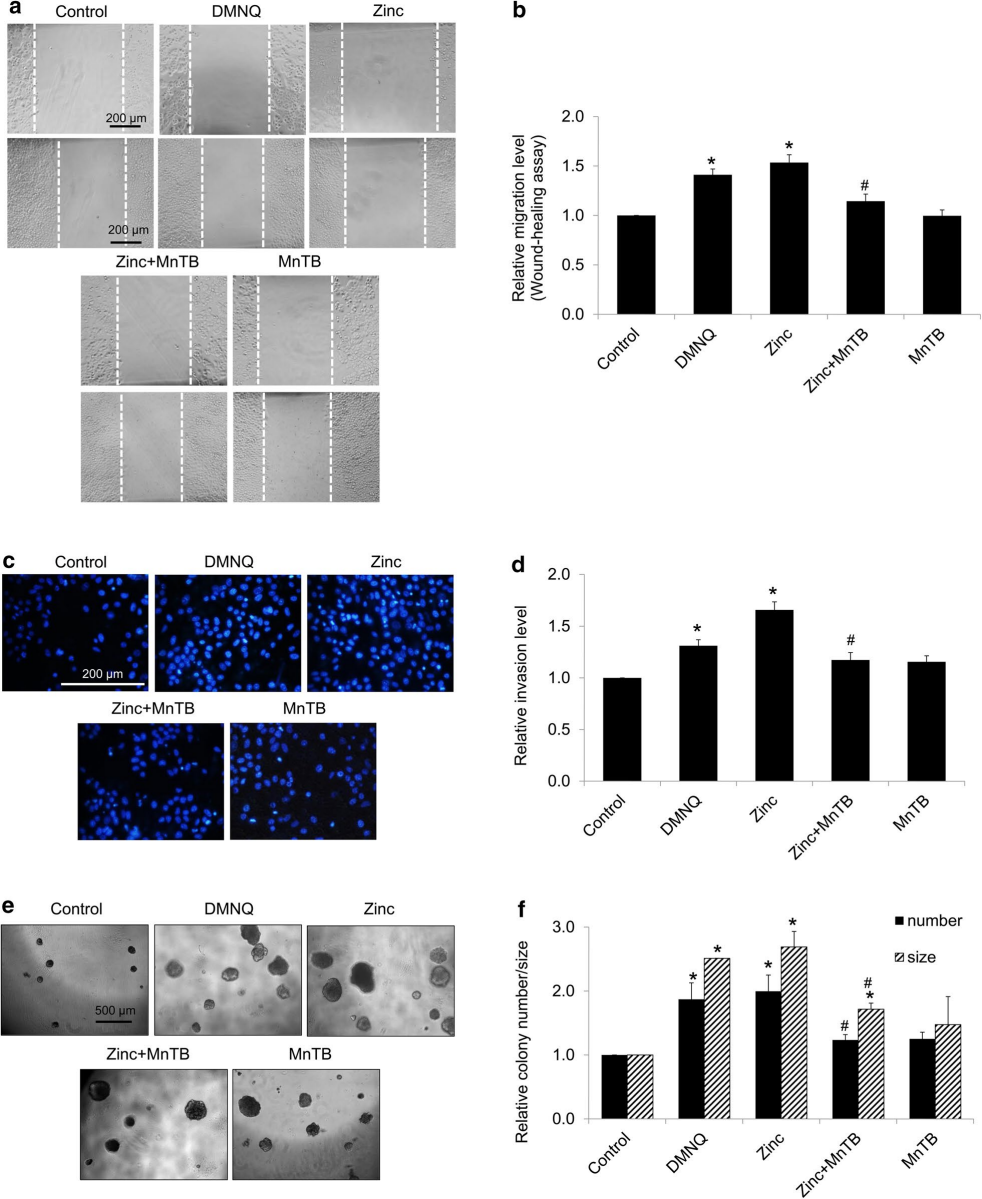
Zinc promotes EMT-related cancer aggressive behaviors of H460 cells through superoxide anion-dependent mechanism. **a** For cell migration, cells were incubated with 5 µM DMNQ (superoxide anion inducer) or 50 µM MnTBAP (superoxide anion inhibitor), in the presence or absence of zinc (50 µM) for 24 h before the assay. After 24 h of incubation, wound spaces were analyzed and represented as a relative migration level. **b** The relative cell migration was determined by comparing the relative change of those in untreated cells. **c**–**d** For invasion, cells were incubated with 5 µM DMNQ or 50 µM MnTBAP in the presence or absence of zinc (50 µM) for 24 h and cells invasion was evaluated using a transwell coated with matrigel as described under “Methods” section. Value was represented as average number of invaded cells in each field relatively to control. **e** For tumorigenicity, cells were incubated with 5 µM DMNQ or 50 µM MnTBAP in the presence or absence of zinc (50 µM) for 24 h prior to grow in matrigel. After 10 days, colonies were visualized under microscope; *scale bar* 500 µm. **f** Value was represented as average diameter and number of colonies in each field relatively to control cells using image analyzer. Values of all experiments are means of independent triplicate experiments ± SD. *p < 0.05 versus untreated control. ^#^p < 0.05 versus 50 µM zinc-treated cells

The migration and invasion of the cells were further evaluated. The results were consistent with the expression levels of EMT proteins that DMNQ and zinc could be able to induce cell migration and invasion and such inductions could be abolished by superoxide anion inhibitor (Fig. 7a–d). Also, the colony size and number which were increased in response to zinc treatment were found to be significantly attenuated by the treatment of MnTBAP (Fig. 7e, f). Taken together, these results pointed out that zinc induces EMT process, migratory behaviors, and tumorigenic potential in the lung cancer cells via superoxide anion-dependent mechanism.

## Discussion

Accumulating data have guided for a long time that zinc, an essential element composition of numerous proteins [6–8, 29], may play important parts on the basis of cancer cell biology. The plasma zinc level was found to be significantly elevated in certain cancer tissues [8–11]. Zinc-containing compounds seem to be associated with carcinogenesis in lung cancer other cancers [30, 31]. In detail, zinc chromate was found to increase cytotoxicity, chromosome damage and DNA double strand breaks in human lung epithelial cells, suggesting the roles of zinc-containing compounds in cell toxicity and carcinogenesis [30]. Besides, the increase of zinc influx transporters such as ZIP6 [12], ZIP7 [13] and ZIP10 [14] has been linked to the aggressive behaviors and poor prognosis of breast cancer [12–14, 32].

Until recently, the knowledge of zinc on the molecular mechanisms of cancer metastasis is still not fully understood especially those regulating EMT. EMT is considered a critical augmenting process of cancer metastasis as it facilitates cancer dissemination in many ways [2–4]. In the process of EMT, cellular phenotypes are altered together with the distinguished expressions of protein markers being changed from epithelial toward mesenchymal types [2–5]. The process facilitates the loss of cell adhesion, increases motility, and survival in detached condition [2–5]. During EMT, an elongated fibroblast-like morphology of the cells is frequently observed. However, indicators like the switching between E-cadherin and N-cadherin, as well as EMT transcription factors snail and slug are more acceptable as hallmarks of EMT [2–5]. In particular, lung cancer H460 cells were shown to undergo EMT in response to various stimuli [33, 34]. EMT features of H460 cells were characterized by (i) the change of cell morphology from epithelial to fibroblast-liked mesenchymal shape, (ii) the increased EMT markers N-cadherin, vimentin, snail, and slug, together with the reduction of epithelial marker E-cadherin, and (iii) EMT behaviors, including increased migration, invasion and tumorigenic potential [33–38]. In consistent with such studies, our results showed that zinc-treated lung cancer cells displayed the elongated mesenchymal-like shape with the significant increase of EMT markers namely N-cadherin, vimentin, snail and slug (Fig. 2). Also, we found that the E-cadherin was dramatically reduced in response to zinc treatment.

Previous studies indicated that EMT facilitates the cell motility by decreasing cell–cell interaction via E-cadherin, while increases in N-cadherin-mediated steady-state of active Rac1 [21, 22]. Similar to N-cadherin, Vimentin was shown to increase FAK and Rac1 activities [23, 24]. We found that zinc could induce EMT, resulting in the increase of cancer cell migration and invasion. The proteins regulating cell motility like FAK, RhoA and Rac1 were found to be activated in response to such up-stream signals (Fig. 3). Also, the EMT event was shown to be a key factor that enhances ability of cancer cells to metastasis by increasing the survival after cell detachment and ability to form tumors [34, 39]. It has been previously reported that the ability of cancer cells in forming new tumor can be enhanced by EMT as a result from snail augmentation [25–27]. We have supported this fact by the demonstration that treatment of the cells with zinc induced EMT with significant increase of snail increased number and size of tumor colonies in 3D culturing anchorage-independent condition (Fig. 4).

In the previous work, we have reported that the widely used chemical agent triclosan could be able to enhance EMT and aggressive behaviors in anoikis-resistant lung cancer cells [34]. However, role of endogenous element zinc on such effects has not been clarified. This current study has reported for the first time that zinc significantly induced EMT and tumorigenic potential in lung cancer cells through the enhancement of cellular superoxide anion level. Superoxide anion has been implicated in various biological and pathological processes [40–42]. Evidence has shown that the level of superoxide anion is frequently upregulated in cancer cells and regulates cancer cell proliferation, migration and metastasis [42–46]. Interestingly, we have first revealed the role of such a specific ROS in regulation of EMT in cancer cells, as the EMT could be induced by addition of superoxide anion generator (Figs. 6, 7). The EMT mediated by zinc treatment was abolished by the superoxide anion inhibitor. These findings not only provide the evidence of endogenous element in regulation of cancer biology, but also add the fact involving specific ROS roles on EMT process of cancer cells. The involvement of ROS signaling on cancer metastasis and EMT has garnered increasing attentions [15–17, 47]. The EMT-related transcription factor snail was shown to be sensitive to the balance of cellular ROS status [47, 48].

Interestingly, zinc was previously addressed to have the potential to interfere with redox status of the cells by inducing oxidative stress [18–20]. In neurons, intracellular zinc is shown to trigger the ROS production during the process of neuron damage [49, 50]. Besides, the elevation of intracellular zinc was shown to induce superoxide anion production from the function of 12-lipoxygenase (12-LOX) enzyme [49, 50]. Interestingly, zinc was demonstrated to promote Hep-2 cancer cell apoptosis by stimulating oxidative stress [18]. Our results showed that zinc could increase superoxide anion in the human lung cancer cells (Fig. 5). The superoxide anion in such cases was suggested to be generated through NADPH oxidase system [19, 20]. Although studies have indicated that hydrogen peroxide can also mediate EMT in human malignant mesothelioma and human ovarian cancer cells [51, 52], treatment of the zinc in our system caused no effect on the cellular level of hydrogen peroxide (Fig. 5). We further investigated the role of superoxide anion on EMT using the superoxide anion inducer DMNQ. DMNQ is known to induce superoxide anion generation via NADPH oxidase activity [53, 54]. We found that treatment of the cells with DMNQ significantly increased the protein hallmarks of EMT as well as metastatic potentials (Figs. 6, 7). Such roles of superoxide anion on EMT were linked with the results of zinc, suggesting that zinc mediates the EMT phenotypes via the production of cellular superoxide anion. These results were confirmed by the ROS inhibitory experiment. Addition of MnTBAP in the zinc-treated cells was shown to abolish superoxide anion induction as well as EMT phenotypes in response to zinc treatment (Figs. 6, 7), strongly indicating that the effect of zinc on EMT was regulated via superoxide-dependent mechanism.

## Conclusions

**Fig. 8:**
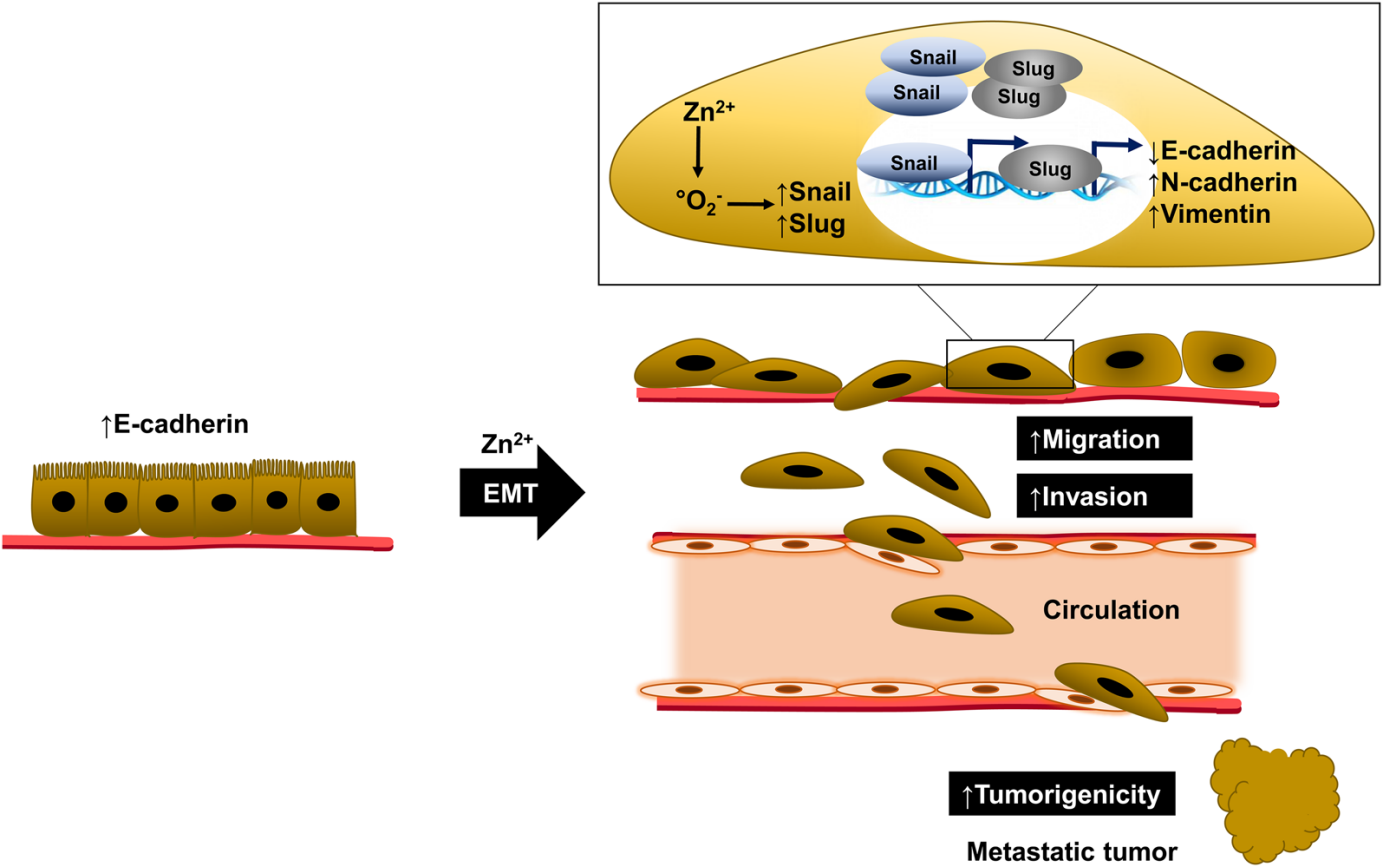
Schematic mechanism of zinc-induced EMT in lung cancer cells. Zinc exposure was found to increase the intracellular superoxide anion and induce EMT phenotypes in lung cancer cells by up-regulating of EMT markers (snail, slug, N-cadherin and vimentin) and down-regulating of E-cadherin protein. Reorganization of adhesion and cytoskeleton proteins resulted in mesenchymal morphology and facilitated aggressive behaviors, including migration, invasion and tumorigenicity in zinc-treated cells. Importantly, zinc-induced lung cancer EMT was clearly inhibited by superoxide anion inhibitor (MnTBAP), suggesting that the induction of the aggressive EMT phenotypes was dependent on zinc-induced superoxide anion generation

In summary, our finding provided the evidence that zinc played a key role in the regulation of EMT and metastatic behaviors. Such inductions of the aggressive EMT phenotypes were dependent on zinc-induced superoxide anion generation as indicated in the summarized schematic figure (Fig. 8). This information helps fulfill the knowledge regarding the role of zinc in tumor cell biology.

## Abbreviations

DCFH_2_-DA: 2,7-dichlorofluorescein diacetate
DHE: dihydroethidium
DMNQ: 2,3-dimethoxy-1,4-naphthoquinone
EMT: epithelial to mesenchymal transition
FAK: focal adhesion kinase
HPF: hydroxyphenyl fluorescein
MnTBAP: Mn (ΙΙΙ) tetrakis (4-benzoic acid) porphyrin chloride
NADPH: nicotinamide adenine dinucleotide phosphate
Rac1: Ras-related C3 botulinum toxin subdtrate 1
RhoA: Ras homolog gene family member A
ROS: reactive oxygen species
ZIP: Zrt/Irt-like protein
